# Hospitalized patients with isolated distal deep vein thrombosis: anticoagulation therapy or not?

**DOI:** 10.1186/s12959-022-00410-1

**Published:** 2022-09-13

**Authors:** Xiaolin Luo, Liying Zhang, Changchun Hou, Pengda Li, Shaofa Wu, Zebi Wang, Enpu Yang, Yun Cui, Ning Sun, Yang Yu, Zhixia An, Jun Jin, Zhexue Qin

**Affiliations:** 1 Department of Cardiology, Xinqiao Hospital, Army Medical University, Shapingba District, No.183, Xinqiaozhengjie Street, Chongqing, China; 2 Department of Cardiovascular Medicine, People’s Hospital of Shapingba District, Chongqing, China; 3 Department of Cardiology, People’s Hospital of Dianjiang County, Chongqing, China; 4 Department of Internal Medicine, Bashan Hospital, Chongqing, China

**Keywords:** Anticoagulation, Isolated distal deep vein thrombosis, Inpatients, Mortality, Pulmonary embolism, Proximal deep vein thrombosis

## Abstract

**Background:**

Isolated distal deep vein thrombosis (IDDVT), a disease frequently detected in hospitalized patients, can progress to proximal deep vein thrombosis (PDVT) and pulmonary embolism (PE). Here, we evaluated the effects of anticoagulation in hospitalized IDDVT patients.

**Methods:**

We conducted a retrospective study in our hospital and enrolled hospitalized IDDVT patients diagnosed by compression ultrasonography (CUS) from January to December 2020. Participants were divided into anticoagulation (AC) and non-anticoagulation (non-AC) groups. After propensity score matching (PSM), multivariate Cox regression analyses were performed to assess whether anticoagulation was associated with PDVT/PE, and all-cause mortality.

**Results:**

A total of 426 IDDVT inpatients with CUS follow-up were screened from 1502 distal DVT patients and finally enrolled. The median age was 67 years with 51.4% males and 15.5% cancer patients. The median follow-up was 11.6 months. There were 288 and 138 patients treated with or without anticoagulants, respectively. Patients in the non-AC group had less body mass index and more comorbidities. Patients in the AC group were treated with rivaroxaban or dabigatran (52.1%), low molecular weight heparin (42.7%), and warfarin (5.2%). The PSM generated 111 pairs of well-matched IDDVT patients with or without anticoagulation. The Kaplan–Meier analysis demonstrated that neither the incidence of PDVT/PE (5.4% vs. 2.7%, log-rank *p* = 0.313) nor all-cause mortality (27.9% vs. 18.9%, log-rank *p* = 0.098) was significant different between groups. Anticoagulation was not associated with PDVT/PE and all-cause mortality in the multivariable Cox regression analyses using the matched cohorts. The main risk factors for all-cause mortality were age, malignancy history, BMI, sepsis, heart failure, and white blood cell (WBC) count.

**Conclusions:**

In hospitalized IDDVT patients, the thrombosis extension rate to PDVT/PE was low. Anticoagulation did not reduce the incidence of thrombosis extension of IDDVT and was not associated with all-cause mortality.

**Supplementary Information:**

The online version contains supplementary material available at 10.1186/s12959-022-00410-1.

## Background

Isolated distal deep vein thrombosis (IDDVT) is defined as distal or calf deep vein thrombosis (DVT) restrained in the infra-popliteal veins of lower limbs without concomitant proximal DVT (PDVT) or pulmonary embolism (PE). IDDVT is a common disease in clinical practice, [1, 2] accounting for 23.4–59.7% of all DVTs [2]. However, the optimal management for IDDVT patients remains controversial. For example, the evidence of anticoagulation in reducing thrombosis extension and recurrence from randomized clinical studies was not consistent in IDDVT patients [3–7]. Therefore, anticoagulation is cautiously recommended by the American College Chest Physicians guidelines for high-risk IDDVT patients, including those with active cancer, a history of venous thromboembolism, and inpatient status [8].

Although patients are hospitalized for different reasons, the inpatient status itself is associated with an increased IDDVT risk [9]. Moreover, the situation of hospitalization, such as acute illness, trauma, and cancer, might also contribute to the incidence of IDDVT [10–12]. Additionally, IDDVT has a thrombosis extension rate of 10% and a PE rate of 1.6–2.6% [13–15]. Besides, inpatients with IDDVT might be more prone to extension or embolization. However, the data on the risk of IDDVT progression is sparse among inpatients. Finally, evidence regarding the efficiency of anticoagulation therapy for IDDVT patients is scarce [16, 17].

Therefore, in the present retrospective study, we investigated the current status of IDDVT management, and whether anticoagulation could affect the outcomes of inpatients, including the rate of thrombosis proximal propagation and all-cause mortality. Overall, we provided an essential reference to guide clinical practice for IDDVT.

## Methods

### Study design

This was a single-center, retrospective study conducted at the Xinqiao Hospital, Army Medical University (Third Military Medical University) in Chongqing, China. We consecutively enrolled patients diagnosed with DDVT by compression ultrasonography (CUS), from January 1^st^ to December 31^st^, 2020. The inclusion criteria were: 1) hospitalized patients with age ≥ 18 years; 2) diagnosed with IDDVT by CUS; 3) with at least one CUS follow-up examination. Patients were excluded if they presented concomitant PDVT or PE, without follow-up CUS, outpatients, significant bleeding at admission, indication for long-term anticoagulation for other reasons, and lost to follow-up. The present study complied with the Declaration of Helsinki and was approved by the ethics committee of the Xinqiao Hospital, Army Medical University (Third Military Medical University). Written consents were waived for its retrospective design by the committee. Patients were divided into two groups, those who received any kind of anticoagulation drugs [anticoagulation (AC) group], and those managed without anticoagulation drugs [non-anticoagulation (non-AC) group].

### Compression ultrasonography (CUS)

Baseline and follow-up ultrasounds were performed at the diagnostic unit or bedside by trained sonographers according to a standardized protocol that included a complete whole-leg ultrasonography [18]. Briefly, patients first laid in the supine position, and the proximal deep veins in the lower extremities were continuously imaged along the veins’ course in the transverse plane with a linear probe (5–10 HZ), including the common femoral, superficial femoral, deep femoral, and popliteal veins. Then, the patients took a seated position with the lower legs hanging down, and the distal deep veins were sequentially examined in this position, including the posterior tibial, peroneal, gastrocnemius, and soleus veins. Anterior tibial veins were not imaged since they were difficult to be compressed and partially obscured by the interosseous membranes and bones. If patients could not change between body positions, CUS was performed just in the supine position. DVT was diagnosed when a filling defect and any lack of compressibility of the deep venous segments were detected. Complete thrombosis resolution during follow-up was defined as no filling defect and complete compressibility restored in segments that were initially involved in the thrombosis by CUS.

### Data collection

The demographic information, medical history, and examination results of participants were collected from electronic medical records. The following data were retrieved: diagnosis date, examination frequencies, and CUS follow-up results. Risk factors for DVT were also documented, including bedridden for more than three days, recent surgery or trauma, pregnancy, family history of venous thromboembolism, congestive heart failure (CHF), stroke, paraplegia, and malignancy history. Other data, such as hypertension, diabetes mellitus (DM), renal insufficiency (RI), hepatic insufficiency (HI), intensive care unit admission (ICU), sepsis, acute myocardial infarction (AMI), and chemo-radiotherapy history, were also collected. The first results of white blood cell (WBC) count, hemoglobin, platelet (PLT) count, serum creatine, and D-dimers at admission were recorded as the baseline data. Meanwhile, the status, drugs, dosage, and duration of anticoagulation therapy during hospitalization and after discharge were also collected.

### Endpoints and follow-up

The primary endpoints of this study were: 1) thrombosis extension into PDVT/PE, and 2) all-cause mortality. Thrombosis extension was defined as imaged-confirmed thrombosis extension to any of the popliteal, femoral, iliac, and cava veins, regardless of whether the patients had clinical symptoms or not. PE was diagnosed according to a recent guideline [19]. The secondary endpoints were: 1) complete thrombosis resolution, and 2) bleeding, including major or minor bleeding events. Participants were followed up through telephone, clinic interviews, and medical records. We also collected CUS data and computed tomography pulmonary angiography from the medical records at other institutions or hospitals, including results during admission, and outpatient visits.

### Statistical analyses

The sample size was calculated using PASS 15 Software (NCSS, LLC. Kaysville, Utah, USA). Referring to the published reports, we assumed the incidence of primary outcome (thrombosis progression to PDVT or PE) is 10% and 3% in IDDVT patients without and with anticoagulation, respectively [5, 7, 20]. The sample size was estimated to be 404 (with 135 in non-AC group and 269 in AC group, respectively), achieving 80% power to detect a difference between the group proportions with significant two-sided level of 0.05.

In unmatched cohorts, continuous variables with normal distribution are presented as means ± standard deviations or medians with interquartile ranges. Comparisons between two samples were performed using t-tests or Wilcoxon rank-sum tests. Categorical variables are presented as frequencies and percentages and were compared with χ^2^ or Fisher’s exact tests.

Propensity-score matching (PSM) was performed according to the method to control the measured confounders between the non-AC and AC groups [21]. Briefly, a propensity score was generated for each patient using multivariable binary logistic regression analysis. Variables with initial clinical relevance and those with statistical significance in the univariate analysis (*p* < 0.10) were included. The non-AC group was matched with the AC group based on the propensity score in a 1:1 ratio, with the Nearest Neighbor Matching algorithm and a caliper of 0.05. After PSM, distribution differences of baseline covariates were assessed through the method proposed by Peter C. Austin and were described as standardized differences [22]. A cutoff < 0.10 indicated well matching between treatment groups.

The time-to-event rates for each group were estimated using the Kaplan–Meier method and compared with log-rank tests in matched cohorts. Moreover, both in unmatched and matched cohorts, a multivariable Cox proportional hazards model analysis was performed to calculate the hazard ratio (HR) and 95% confidence intervals (CIs) to explore the association of anticoagulation and other risks factors with the primary endpoints. Variables considered to be clinically relevant or that showed statistical significance in the univariable analysis (*p* < 0.10) with the primary endpoints were included in the multivariable regression model. A *p* < 0.05 indicated significant differences between measurements. All analyses were performed using SPSS version 22.0 (IBM Corp., Armonk, NY, USA), and the PSM also required the R software (v. 2.15.3, R Foundation for Statistical Computing), SPSS R plug-in (SPSS Statistics Essentials for R 22.0.0, IBM, USA), and PS Matching in SPSS (version 3.04, SourceForge, San Diego, USA).

## Results

### Baseline characteristics

**Fig. 1:**
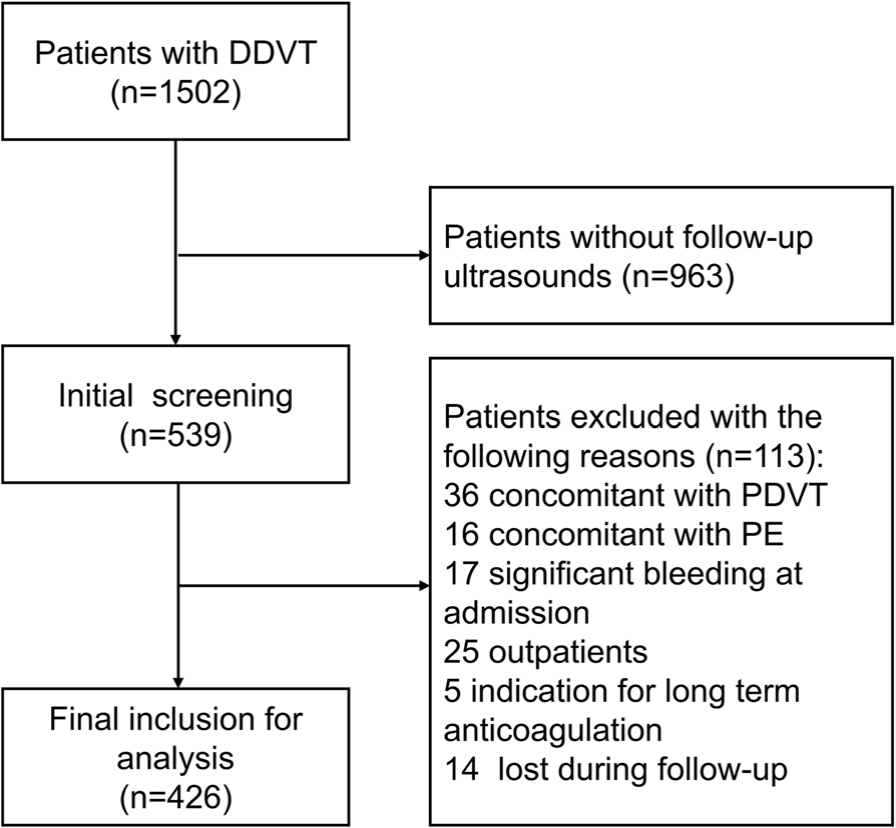
Flow diagram of patients’ enrollment. DDVT, distal deep venous thrombosis; PDVT, proximal deep venous thrombosis; PE, pulmonary embolism

From January to December 2020, 1502 patients were diagnosed with DDVT by CUS, including patients with clinical suspicion of DVT or PE. A total of 963 patients underwent just one CUS examination and were excluded. Further, 113 patients were excluded for other reasons, including concomitant PDVT (*n* = 36) or PE (*n* = 16), significant bleeding at admission (*n* = 17), outpatients (*n* = 25), anticoagulation therapy for other causes (*n* = 5), and lost during follow-up (*n* = 14). Finally, 426 inpatients diagnosed with IDDVT were included for analysis (Fig. 1). The median age was 67 years, and 51.4% were males. Among participants, 43.0% and 17.6% had hypertension and DM, respectively. Moreover, 66 patients presented a malignancy history and 248 underwent surgeries for different reasons. The median follow-up time was 11.6 months. Additionally, 138 patients did not receive anticoagulation drugs, while the other 288 IDDVT patients underwent anticoagulation therapy. Compared to the AC group (*n* = 288), patients in the non-AC group (*n* = 138) had lower body mass index (BMI) and were less likely to undergo surgeries. Meanwhile, patients in the non-AC group had more comorbidities, including hypertension, DM, RI, and stroke. Detailed baseline characteristics are displayed in Table 1.

**Table 1:** Baseline characteristics in unmatched and matched cohorts

	**Unmatched**				**PSM**^a^		
	**non-AC *n* = 138**	**AC *n* = 288**	**p**	**SD1**	**non-AC *n* = 111**	**AC *n* = 111**	**SD2**
**Age, year**	67 (57–72)	67.5 (56.25–75.00)	0.500	0.036	67 (57–71)	67 (56–73)	-0.033
**BMI, kg/m**^**2**^	22.5 (20.5–25.4)	24.03 (21.4–26.1)	0.012	0.228	22.5 (20.5–25.6)	23.5 (20.0–25.4)	-0.052
**Male, n (%)**	84 (60.9)	135 (46.9)	0.007	-0.284	64 (57.7)	60 (54.1)	-0.073
**HTN, n (%)**	68 (49.3)	115 (39.9)	0.068	-0.190	49 (44.1)	52 (46.8)	0.054
**DM, n (%)**	34 (24.6)	41 (14.2)	0.008	-0.265	20 (18.0)	19 (17.1)	-0.024
**RI, n (%)**	22 (15.9)	28 (9.7)	0.062	-0.186	17 (15.3)	15 (13.5)	-0.051
**HI, n (%)**	17 (12.3)	24 (8.3)	0.192	-0.132	12 (10.8)	13 (11.7)	0.028
**Bedridden** **(≥ 3 days), n (%)**	94 (68.1)	171 (59.4)	0.082	-0.182	70 (63.1)	70 (63.1)	0.000
**ICU, n (%)**	65 (47.1)	100 (34.7)	0.014	-0.254	46 (41.4)	49 (44.1)	0.055
**Sepsis, n (%)**	5 (3.6)	9 (3.1)	0.787	-0.028	4 (3.6)	7 (6.3)	0.125
**AMI, n (%)**	3 (2.2)	8 (2.8)	0.713	0.038	3 (2.7)	6 (5.4)	0.137
**CHF, n (%)**	7 (5.1)	13 (4.5)	0.799	-0.028	5 (4.5)	6 (5.4)	0.042
**Stroke, n (%)**	45 (32.6)	50 (17.4)	0.000	-0.357	27 (24.3)	22 (19.8)	-0.109
**Paraplegia, n (%)**	2 (1.4)	7 (2.4)	0.724	0.073	2 (1.8)	3 (2.7)	0.061
**Malignancy history, n (%)**	21 (15.2)	45 (15.6)	0.967	0.011	19 (17.1)	20 (18.0)	0.024
**Chemo-radiotherapy, n (%)**	7 (5.1)	16 (5.6)	0.836	0.022	6 (5.4)	5 (4.5)	-0.042
**VTE history, n (%)**	0 (0.0)	0 (0.0)	-	0.000	0 (0.0)	0 (0.0)	0.000
**Pregnancy, n (%)**	0 (0.0)	1 (0.3)	1.000	0.078	0 (0.0)	0 (0.0)	0.000
**Surgery, n (%)**	56 (40.6)	192 (66.7)	0.000	0.542	53 (47.7)	53 (47.7)	0.000
**WBC, 10**^**9**^**/L**	8.70 (6.52–11.01)	7.46 (5.75–10.46)	0.036	-0.112	8.64 (6.37–10.79)	7.94 (5.79–10.80)	-0.041
**HG, g/L**	109 (95–126)	115 (99–128)	0.130	0.127	109 (96–124)	114 (96–126)	0.012
**PLT, 10**^**9**^**/L**	200 (146–265)	189 (148–263)	0.983	0.012	201 (146–270)	191 (151–268)	0.026
**CREA, μmol/L**	65.8 (55.0–79.8)	68.6 (55.4–84.9)	0.465	0.001	66.0 (54.6–78.5)	69.7 (55.6–87.3)	0.113
**D-dimer, mg/L**	0.99 (0.54–2.99)	1.04 (0.48–2.84)	0.682	0.015	1.00 (0.54–2.99)	1.19 (0.57–2.87)	0.111

*BMI* Body mass index, *AC* Anticoagulation, *SD* Standardized difference, *HTN* Hypertension, *DM* Diabetes mellitus, *RI* Renal insufficiency, *HI* Hepatic insufficiency, *ICU* Intensive care unit, *AMI* Acute myocardial infarction, *CHF* Congestive heart failure, *VTE* Venous thromboembolism, *WBC* White blood cell, *HG* Hemoglobin, *PLT* Platelet, *CREA* Creatine, *PSM* Propensity score matching

^a^Propensity score of each patient was calculated by binary logistic regression model, treatment type (anticoagulation or not) was considered as dependent variable, and the independent variables contained age, male, BMI, HTN, DM, RI, HI, bedridden (≥ 3 days), ICU, stroke, malignancy history, surgery, WBC, and HG

In the AC group, patients received different anticoagulation drugs: 52.1% took dabigatran etexilate or rivaroxaban, 42.7% received low molecular weight heparin (LMWH) injection, and 5.2% took warfarin. The median duration of anticoagulation therapy was 0.61 months. The number of patients with anticoagulation duration ≤ 1, 1–3, and > 3 months was 192 (66.7%), 43 (14.9%), and 56 (19.4%), respectively.

After PSM, 111 pairs of IDDVT patients were well matched, with variables including age, male, BMI, HTN, DM, RI, HI, bedridden, ICU, stroke, malignancy history, surgery, WBC count, and HG. Although some variables were not included in the propensity score calculation, the standardized differences of the unbalanced variables before the PSM were minimized through the PSM.

### Primary outcomes

The endpoint results are presented in Table 2. Patients in the AC group who progressed to DVT/PE were not statistically different from those in the non-AC group (4.9% vs. 5.1%, *p* = 0.925). Specifically, the incidence of PE in both groups was low and no difference was observed between the groups (0.7% vs. 1.4%, *p* = 1.000). Patients who progressed to PDVT in the AC group did not differ from those in the non-AC group (4.2% vs. 5.1%, *p* = 0.672). After PSM, these observations remained robust.

**Table 2:** Primary and secondary endpoints in patients with or without anticoagulation in unmatched and matched cohorts

	**Unmatched**			**PSM**		
	**non-AC *n* = 138**	**AC *n* = 288**	**p1**	**non-AC *n* = 111**	**AC *n* = 111**	**p2**
**Primary endpoint**						
**PDVT/ PE, n (%)**	7 (5.1)	14 (4.9)	0.925	6 (5.4)	3 (2.7)	0.499
**PDVT, n (%)**	7 (5.1)	12 (4.2)	0.672	6 (5.4)	3 (2.7)	0.499
**PE, n (%)**	1 (0.7)	4 (1.4)	1.000	1 (0.9)	0 (0.0)	1.000
**All-cause mortality, n (%)**	41 (29.7)	47 (16.3)	0.001	31 (27.9)	21 (18.9)	0.113
**Thrombosis related death, n (%)**	0 (0.0)	0 (0.0)	-	0 (0.0)	0 (0.0)	-
**Cardiovascular death, n (%)**	4 (2.9)	8 (2.8)	1.000	2 (1.8)	3 (2.7)	0.651
**Secondary endpoint**						
**Thrombosis resolution, n (%)**	58 (42.0)	155 (53.8)	0.023	48 (43.2)	57 (51.4)	0.226
**Bleeding, n (%)**	9 (6.5)	8 (2.8)	0.065	6 (5.4)	5 (4.5)	0.757

*AC* Anticoagulation, *PDVT* Proximal deep venous thrombosis, *PE* Pulmonary embolism, *PSM* Propensity score matching

**Fig. 2:**
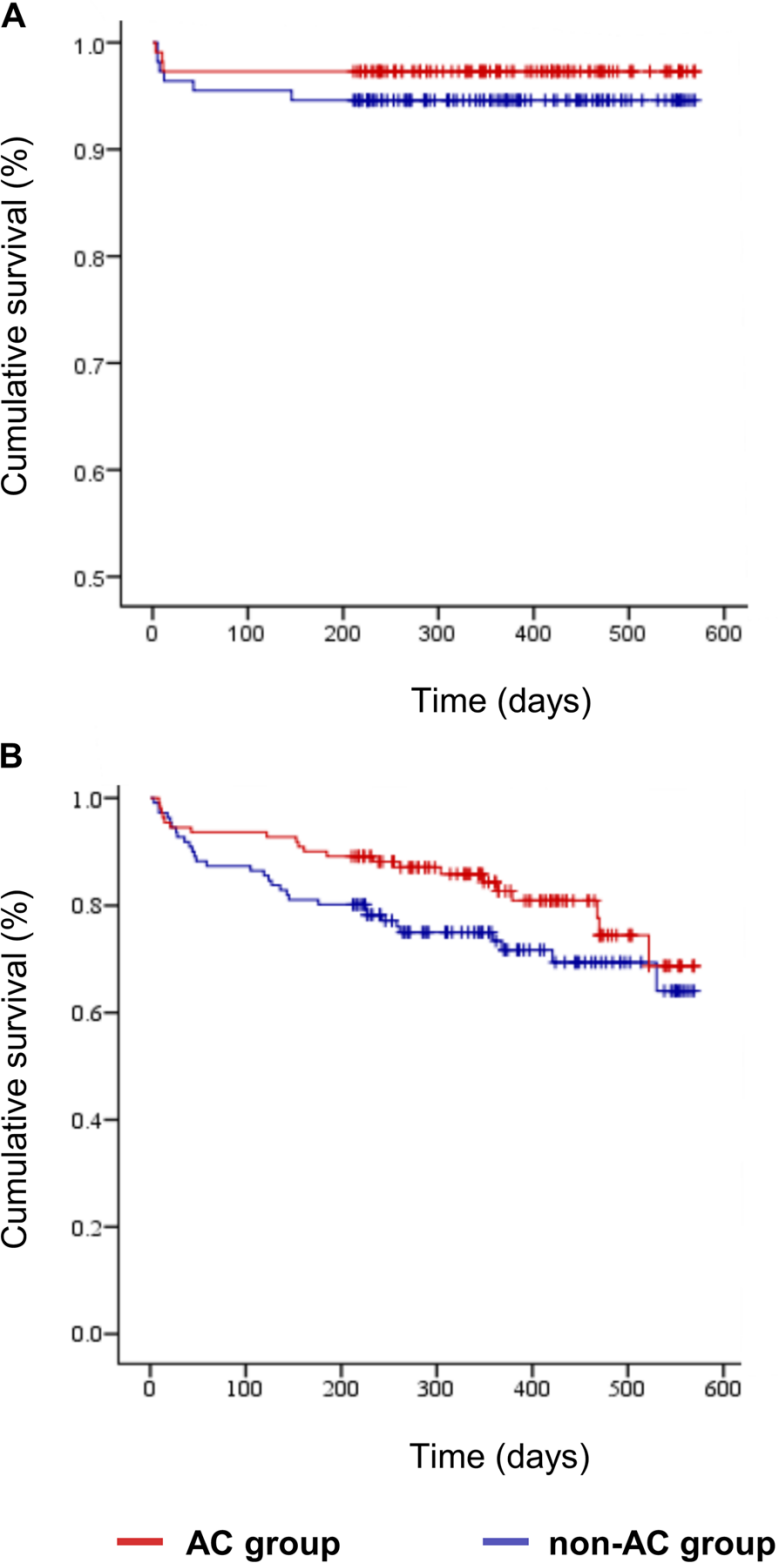
Kaplan–Meier analyses of primary endpoints with anticoagulation in matched cohorts. **A** PDVT/PE in hospitalized IDDVT patients with or without anticoagulation. (non-AC group vs. AC group, 5.4% vs. 2.7%, log-rank *p* = 0.313). **B** All-cause mortality in hospitalized IDDVT patients with or without anticoagulation. (non-AC group vs. AC group, 27.9% vs. 18.9%, log-rank *p* = 0.098). AC, anticoagulation

Regarding mortality, there was no thrombosis-related death in both groups. The cardiovascular deaths were not different between groups (2.9% vs. 2.8%, *p* = 1.000). Deaths were mostly attributed to the original diseases (see Additional file 1). Before PSM, patients in the non-AC group had higher all-cause mortality than those in the AC group (29.7% vs. 16.3%, *p* = 0.001). After PSM, the Kaplan–Meier analysis demonstrated that neither the incidence of PDVT/PE (5.4% vs. 2.7%, log-rank *p* = 0.313) nor all-cause mortality (27.9% vs. 18.9%, log-rank *p* = 0.098) were significantly different between groups (Fig. 2).

### Secondary outcomes

Patients in the AC group had a higher percentage of complete thrombosis resolution (53.8% vs. 42.0%, *p* = 0.023). The bleeding was similar in the two groups (AC group 2.8% vs. non-AC group 6.5%, *p* = 0.065). However, the thrombosis resolution and bleeding were not different between groups after PSM.

### Risk factors of thrombosis extension and all-cause mortality

The univariable and multivariable Cox regression analyses showed that anticoagulation was not associated with thrombosis extension. Furthermore, none of the documented variables were independently related to thrombosis progression (see Additional file 2).

In the unmatched cohort, we found that anticoagulation was associated with lower all-cause mortality (HR = 0.580, 95% CI 0.376–0.895, *p* = 0.014) in multivariable COX regression analysis. However, this association diminished after PSM. The factors related to all-cause mortality were age, BMI, sepsis, heart failure, malignancy history, and WBC count after PSM (Table 3).

**Table 3:** Multivariable Cox regression analyses to estimate the factors associated with all-cause mortality in unmatched and matched cohorts

	**Unmatched**			**PSM**		
	**HR**	**95%CI**	**p1**	**HR**	**95 CI**	**p2**
**Age**	1.033	1.012–1.054	0.002	1.037	1.010–1.064	0.007
**BMI**	-	-	-	1.090	1.005–1.182	0.038
**Sepsis**	2.857	1.273–6.411	0.011	3.005	1.149–7.859	0.025
**CHF**	-	-	-	3.492	1.290–9.455	0.014
**Malignancy history**	3.760	2.321–6.091	0.000	3.651	1.984–6.718	0.000
**Surgery**	0.530	0.339–0.829	0.005	-	-	-
**Anticoagulation**	0.580	0.376–0.895	0.014	-	-	-
**WBC**	1.066	1.019–1.114	0.005	1.078	1.014–1.147	0.016

*BMI* Body mass index, *CHF* Congestive heart failure, *WBC* White blood cell, *HR* Hazard ratio, *CI* Confidence interval, *PSM* Propensity score matching

## Discussion

In the present study, we found that anticoagulation did not reduce the thrombosis extension in IDDVT inpatients, nor it was associated with their all-cause mortality. The main risk factors for all-cause mortality were age, BMI, sepsis, CHF, malignancy history, and WBC count.

Previous studies have suggested that IDDVT patients have a lower incidence of thrombosis recurrence and all-cause mortality than PDVT patients [9, 16, 23, 24]. Moreover, the risk of thrombosis extension of IDDVT is around 3–10% when left untreated [13–15, 25]. This was also reflected in our current study, which showed that the incidence of proximal propagation rate was 5.1%. Considering the low embolic risk of IDDVT, diagnosis and anticoagulation therapy for IDDVT were not associated with better outcomes [18, 26]. Hence, the necessity of anticoagulation therapy for IDDVT patients was questioned. Randomized, double-blind, and placebo-controlled studies have demonstrated no efficacy of anticoagulation in reducing thrombosis extension and PE, but increased bleeding in low-risk outpatients [7].

Furthermore, hospitalization is a risk factor for IDDVT occurrence. Previous evidence has shown that hospitalization for medical diseases, [12, 27] trauma, [11, 23] major surgery, [23, 28] and critical status [10] are independent risk factors for thrombosis formation. Additionally, Heit et al. found that inpatients status had more than 100 times increased incidence of thrombosis formation, including IDDVT, compared to residents in the communities [29]. Anticoagulation is empirically prescribed by clinicians based on the concerns of embolic risk and the recommendation for DVT. However, whether anticoagulation in hospitalized patients can reduce the proximal propagation of IDDVT remains controversial. For example, Giovanna et al. found that anticoagulation could not decrease the risk of thrombosis propagation to proximal deep veins among hospitalized IDDVT patients [16]. Moreover, Yorinari et al. retrospectively found that anticoagulation therapy for hospitalized IDDVT patients had no effects on the incidence of PE while increased bleeding events [17]. Similarly, we showed that the incidence of PE was 0.7% among IDDVT patients and anticoagulation did not reduce the incidence of PE, consistent with previous studies [24, 30]. Besides, inpatient status might increase the prothrombotic risk factor in IDDVT patients but anticoagulation failed to reduce the proximal propagation in these patients.

The association between coagulation and mortality is another concern in IDDVT patients. Previously, Giovanna’s group showed that anticoagulation might reduce all-cause mortality of inpatients [16]. Herein, we did not observe this effect. The disagreements might lie in the population heterogeneity and the different adjustment of confounders. Further, we found that the deaths were attributed to the original diseases rather than other PDVT/PE propagated by IDDVT. Meanwhile, another IDDVT cohort showed that 1.2% of deaths were attributable to PE, including comorbidities such as cancer [20]. This observation was consistent with our current study. Particularly, the factors most related to death were age, BMI, sepsis, CHF, malignancy history, and WBC count. Interestingly, malignancy history was the most important risk factor for death. Previously, it was well-established that cancer was the main reason for death in IDDVT patients during long-term follow-up [9, 16, 31, 32].

## Limitation

Our current study also has some limitations. First, this was a retrospective single-center study, which might lead to intrinsic bias, including selection and measurement bias. Hence, we consecutively included patients diagnosed with IDDVT in our hospital during 2020 to reduce the selection bias. Meanwhile, we used multivariate Cox regression analysis and PSM to balance the difference between groups and adjust the confounders. Second, patients were not regularly scheduled for follow-ups, which might result in the underestimation of primary endpoints, especially the proximal progression to PDVT or PE. The recall bias of endpoints from the participants might also exist. Thus, we tried to retrieve the CUS results performed in our institution and other hospitals if the patients had undergone the CUS examination. Third, as we know, thrombophilic predisposition, such as antiphospholipid antibodies, protein C, protein S or antithrombin deficiency, are risk factors of venous thromboembolism development and recurrence. Whether they are associated with IDDVT progression to PDVT or PE is elusive. Unfortunately, we are unable to provide the anticoagulation data of these patients because of the retrospective design. Last, Given the incidence of PDVT/PE in patients without anticoagulation was lower than assumed, it is possible that the sample size of our study may be underpowered to verify the proportion difference between the groups. Further study with a larger population might be required.

## Conclusion

In the present study, we demonstrated that the incidence of thrombosis propagation to PDVT or PE was low in hospitalized IDDVT patients, and did not identify risk factors associated with thrombosis extension. Anticoagulation did not lead to decreasing extension to PDVT/PE or all-cause mortality. Finally, future well-designed prospective studies on IDDVT are still required.

## Supplementary Information

**Additional file 1:** **Supplementary Table 1.** Causes of death in patients with and without anticoagulation.

**Additional file 2:** **Supplementary Table 2.** Univariate Cox regression analyses to estimate the factors associated with PDVT/ PE.

## Data Availability

The datasets used and/or analyzed during the current study are available from the corresponding author on reasonable request.

## Abbreviations

AC: Anticoagulation
non-AC: Non-anticoagulation
AMI: Acute myocardial infarction
BMI: Body mass index
CHF: Congestive heart failure
CIs: Confidence intervals
CUS: Compression ultrasonography
DM: Diabetes mellitus
DVT: Deep venous thrombosis
HI: Hepatic insufficiency
HR: Hazard ratio
IDDVT: Isolated distal deep venous thrombosis
ICU: Intensive care unit
LMWH: Low molecular weight heparin
PDVT: Proximal deep venous thrombosis
PE: Pulmonary embolism
PLT: Platelet
PSM: Propensity-score matching
RI: Renal insufficiency
WBC: White blood cell

## References

[1] Johnson SA, Stevens SM, Woller SC, Lake E, Donadini M, Cheng J (2010). Risk of deep vein thrombosis following a single negative whole-leg compression ultrasound: a systematic review and meta-analysis. JAMA.

[2] Palareti G, Schellong S (2012). Isolated distal deep vein thrombosis: what we know and what we are doing. J Thromb Haemost.

[3] Pinede L, Ninet J, Duhaut P, Chabaud S, Demolombe-Rague S, Durieu I (2001). Comparison of 3 and 6 months of oral anticoagulant therapy after a first episode of proximal deep vein thrombosis or pulmonary embolism and comparison of 6 and 12 weeks of therapy after isolated calf deep vein thrombosis. Circulation.

[4] Lagerstedt CI, Olsson CG, Fagher BO, Oqvist BW, Albrechtsson U (1985). Need for long-term anticoagulant treatment in symptomatic calf-vein thrombosis. Lancet.

[5] Horner D, Hogg K, Body R, Nash MJ, Baglin T, Mackway-Jones K (2014). The anticoagulation of calf thrombosis (ACT) project: results from the randomized controlled external pilot trial. Chest.

[6] Schwarz T, Buschmann L, Beyer J, Halbritter K, Rastan A, Schellong S (2010). Therapy of isolated calf muscle vein thrombosis: a randomized, controlled study. J Vasc Surg.

[7] Righini M, Galanaud JP, Guenneguez H, Brisot D, Diard A, Faisse P (2016). Anticoagulant therapy for symptomatic calf deep vein thrombosis (CACTUS): a randomised, double-blind, placebo-controlled trial. Lancet Haematol.

[8] Kearon C, Akl EA, Ornelas J, Blaivas A, Jimenez D, Bounameaux H (2016). Antithrombotic Therapy for VTE Disease: CHEST Guideline and Expert Panel Report. Chest.

[9] Galanaud JP, Quenet S, Rivron-Guillot K, Quere I, Sanchez Munoz-Torrero JF, Tolosa C (2009). Comparison of the clinical history of symptomatic isolated distal deep-vein thrombosis vs. proximal deep vein thrombosis in 11 086 patients. J Thromb Haemost.

[10] Boddi M, Peris A (2017). Deep Vein Thrombosis in Intensive Care. Adv Exp Med Biol.

[11] Bendinelli C, Balogh Z (2008). Postinjury thromboprophylaxis. Curr Opin Crit Care.

[12] Samama MM, Cohen AT, Darmon JY, Desjardins L, Eldor A, Janbon C (1999). A comparison of enoxaparin with placebo for the prevention of venous thromboembolism in acutely ill medical patients Prophylaxis in Medical Patients with Enoxaparin Study Group. N Engl J Med.

[13] Righini M, Paris S, Le Gal G, Laroche JP, Perrier A, Bounameaux H (2006). Clinical relevance of distal deep vein thrombosis Review of literature data. Thromb Haemost.

[14] Palareti G, Cosmi B, Lessiani G, Rodorigo G, Guazzaloca G, Brusi C (2010). Evolution of untreated calf deep-vein thrombosis in high risk symptomatic outpatients: the blind, prospective CALTHRO study. Thromb Haemost.

[15] Spencer FA, Kroll A, Lessard D, Emery C, Glushchenko AV, Pacifico L (2012). Isolated calf deep vein thrombosis in the community setting: the Worcester Venous Thromboembolism study. J Thromb Thrombolysis.

[16] Elmi G, Aluigi L, Allegri D, Rinaldi ER, Camaggi V, Di Giulio R (2020). Calf deep vein thrombosis: frequency, therapeutic management, early outcomes and all-causes mortality in a cohort of hospitalized patients. Int Angiol.

[17] Ochiai Y, Yamaguchi T, Komiyama C, Kodama T (2021). Impact of Anticoagulation Therapy on the Risk of Pulmonary Embolism and Bleeding Events in Patients with Isolated Distal Deep-Vein Thrombosis. Int Heart J.

[18] Bernardi E, Camporese G, Buller HR, Siragusa S, Imberti D, Berchio A (2008). Serial 2-point ultrasonography plus D-dimer vs whole-leg color-coded Doppler ultrasonography for diagnosing suspected symptomatic deep vein thrombosis: a randomized controlled trial. JAMA.

[19] Konstantinides SV, Meyer G, Becattini C, Bueno H, Geersing GJ, Harjola VP (2019). 2019 ESC Guidelines for the diagnosis and management of acute pulmonary embolism developed in collaboration with the European Respiratory Society (ERS): The Task Force for the diagnosis and management of acute pulmonary embolism of the European Society of Cardiology (ESC). Eur Respir J.

[20] Utter GH, Dhillon TS, Salcedo ES, Shouldice DJ, Reynolds CL, Humphries MD (2016). Therapeutic Anticoagulation for Isolated Calf Deep Vein Thrombosis. JAMA Surg.

[21] Huang F, Du C, Sun M, Ning B, Luo Y, An S (2015). Propensity score matching in SPSS. Nan Fang Yi Ke Da Xue Xue Bao = J Southern Medical University.

[22] Austin PC (2009). Balance diagnostics for comparing the distribution of baseline covariates between treatment groups in propensity-score matched samples. Stat Med.

[23] Schellong SM, Goldhaber SZ, Weitz JI, Ageno W, Bounameaux H, Turpie AGG (2019). Isolated Distal Deep Vein Thrombosis: Perspectives from the GARFIELD-VTE Registry. Thromb Haemost.

[24] Galanaud JP, Sevestre MA, Genty C, Kahn SR, Pernod G, Rolland C (2014). Incidence and predictors of venous thromboembolism recurrence after a first isolated distal deep vein thrombosis. J Thromb Haemost.

[25] Macdonald PS, Kahn SR, Miller N, Obrand D (2003). Short-term natural history of isolated gastrocnemius and soleal vein thrombosis. J Vasc Surg.

[26] Gibson NS, Schellong SM, Kheir DY, Beyer-Westendorf J, Gallus AS, McRae S (2009). Safety and sensitivity of two ultrasound strategies in patients with clinically suspected deep venous thrombosis: a prospective management study. J Thromb Haemost.

[27] Leizorovicz A, Cohen AT, Turpie AG, Olsson CG, Vaitkus PT, Goldhaber SZ (2004). Randomized, placebo-controlled trial of dalteparin for the prevention of venous thromboembolism in acutely ill medical patients. Circulation.

[28] Vlazny DT, Pasha AK, Kuczmik W, Wysokinski WE, Bartlett M, Houghton D (2021). Outcome of anticoagulation in isolated distal deep vein thrombosis compared to proximal deep venous thrombosis. J Thromb Haemost.

[29] Heit JA, Melton LJ, Lohse CM, Petterson TM, Silverstein MD, Mohr DN (2001). Incidence of venous thromboembolism in hospitalized patients vs community residents. Mayo Clin Proc.

[30] Nitta D, Mitani H, Ishimura R, Moriya M, Fujimoto Y, Ishiwata S (2013). Deep vein thrombosis risk stratification. Int Heart J.

[31] Galanaud JP, Sevestre-Pietri MA, Bosson JL, Laroche JP, Righini M, Brisot D (2009). Comparative study on risk factors and early outcome of symptomatic distal versus proximal deep vein thrombosis: results from the OPTIMEV study. Thromb Haemost.

[32] Galanaud JP, Sevestre MA, Pernod G, Genty C, Richelet S, Kahn SR (2017). Long-term outcomes of cancer-related isolated distal deep vein thrombosis: the OPTIMEV study. J Thromb Haemost.

