# Supplementary material for: Hospitalized patients with isolated distal deep vein thrombosis: anticoagulation therapy or not?
Source: Thromb J. 2022 Sep 13;20:52. doi: 10.1186/s12959-022-00410-1 (PMC9472408; doi:10.1186/s12959-022-00410-1)
Supplement: Supplementary file 1 — Additional file 1: Supplementary Table 1. Causes of death in patients with and without anticoagulation. [file 12959_2022_410_MOESM1_ESM.docx]

|  | Total | non-AC | AC | p |
| --- | --- | --- | --- | --- |
| Reasons of death, n (%) | 88(100) | 41(100) | 47(100) | 0.549 |
| Cancer, n (%) | 24(27.3) | 13(31.7) | 11(23.4) |  |
| Stoke ^a^, n (%) | 25(28.4) | 14(34.1) | 11(23.4) |  |
| Cardiovascular diseases, n (%) | 12(13.6) | 4(9.8) | 8(17.0) |  |
| Infections, n (%) | 16(18.2) | 7(17.1) | 9(19.1) |  |
| Others, n (%) | 4(4.5) ^b^ | 1(2.4) | 3(6.4) |  |
| Unknown, n (%) | 7(8.0) | 2(4.9) | 5(10.6) |  |

**Supplementary Table 1.** Causes of death in patients with and without anticoagulation.

AC, anticoagulation.

a, including ischemic and hemorrhagic stoke

b, including two metabolic encephalopathy, one epilepsy, and one Guillain-Barre syndrome.
