# Supplementary material for: Hospitalized patients with isolated distal deep vein thrombosis: anticoagulation therapy or not?
Source: Thromb J. 2022 Sep 13;20:52. doi: 10.1186/s12959-022-00410-1 (PMC9472408; doi:10.1186/s12959-022-00410-1)
Supplement: Supplementary file 2 — Additional file 2: Supplementary Table 2. Univariate Cox regression analyses to estimate the factors associated with PDVT/ PE. [file 12959_2022_410_MOESM2_ESM.docx]

**Supplementary Table 2.** Univariate Cox regression analyses to estimate the factors associated with PDVT/ PE.

|  | **HR** | **95%CI** | **p** |
| --- | --- | --- | --- |
| Age | 0.992 | 0.958-1.027 | 0.636 |
| BMI | 1.035 | 0.925-1.158 | 0.549 |
| Male | 1.385 | 0.902-2.125 | 0.137 |
| HTN | 1.092 | 0.717-1.663 | 0.682 |
| DM | 1.033 | 0.350-3.053 | 0.953 |
| RI | 1.714 | 0.580-5.064 | 0.330 |
| HI | 0.973 | 0.227-4.169 | 0.971 |
| Bedridden (≥3 days) | 1.306 | 0.533-3.204 | 0.559 |
| ICU | 1.123 | 0.480-2.628 | 0.789 |
| Sepsis | 3.240 | 0.754-13.914 | 0.114 |
| AMI | 0.048 | 0.000-4656.472 | 0.604 |
| CHF | 0.918 | 0.123-6.826 | 0.933 |
| Stroke | 0.527 | 0.156-1.784 | 0.303 |
| Paraplegia | 0.532 | 0.074-3.819 | 0.530 |
| Malignancy history | 0.867 | 0.257-2.932 | 0.819 |
| Chemo-radiotherapy | 0.804 | 0.108-5.982 | 0.832 |
| Surgery | 0.603 | 0.261-1.397 | 0.238 |
| Anticoagulation | 0.848 | 0.356-2.022 | 0.710 |
| WBC | 1.04 | 0.947-1.141 | 0.413 |
| HG | 0.992 | 0.973-1.010 | 0.373 |

PDVT, proximal deep vein thrombosis; PE, pulmonary embolism; BMI, body mass index; HTN, hypertension; DM, diabetes mellitus; RI, renal insufficiency; HI, hepatic insufficiency; ICU, intensive care unit; AMI, acute myocardial infarction; CHF, congestive heart failure; WBC, white blood cell; HG, hemoglobin; HR, hazard ratio; CI, confidence interval.
